# Identification of mutations in bone morphogenetic protein 2, 4 and 7 in congenital heart disease patients from Indian population

**DOI:** 10.1186/1755-8166-7-S1-P32

**Published:** 2014-01-21

**Authors:** Ritu Bhardwaj, Damyanti Agrawal, Ashok Kumar, Bhagyalaxmi Mohapatra

**Affiliations:** 1 Cytogenetics Laboratory, Department of Zoology, Banaras Hindu University, Varanasi, India; 2 Department of Cardio Thoracic & Vascular Surgery, Banaras Hindu University, Varanasi, India; 3 Department of Pediatric Medicine, Institute of Medical Sciences, Banaras Hindu University, Varanasi, India

## Background

Congenital heart disease (CHD) is the most common congenital defect, affecting 1% of all livebirths. Initial cardiac development and the subsequent maturation of the heart are temporally and spatially regulated by several Bone Morphogenetic Proteins (BMPs). BMP signaling plays an important role in the specification and patterning of the early embryo. Animal model studies have established a strong link between BMPs and cardiac development. At least 6 BMPs (BMP2, BMP4, BMP5, BMP6, BMP7 & BMP10) are expressed in the heart where they have both independent and redundant functions. BMP2, 4 & 7 are expressed in the AtrioVentricular cushion and Out Flow Tract (OFT) and helps in their formation. However due to functional redundancy of BMP ligands, relatively little is known about the role of individual BMPs. The association of BMPs with CHD has not been studied so far in human subjects. Here we report the genetic variants in BMP2 (NM_001200.2), BMP4 (NM_001202.3) and BMP7 (NM_001719.2) in individuals with CHD.

## Materials & methods

We screened 190 unrelated probands with CHD and 100 healthy controls by PCR and DNA sequencing for 3 genes.

## Results & conclusion

We have identified 6 novel sequence variants in BMP2. Out of which 3 (H321L, E328K & S351C) are missense variants. One nonsense variants (K241X) has also been identified in one case having Patent Ductus Arteriosus and is not found in 100 controls. In BMP7, 10 novel sequence variants have been identified, out of which 3 (D85V, R175W & N372Y) are missense, 3 (c.366G>A, c.381G>A & c.384C>T) are in 5’UTR, 1 synonymous change (c.682G>T) and 3 (c.947-20G>C, c.1140+14G>A & c.1289+68G>A) are intronic variants. In BMP4, 3 sequence variants are found, out of which only one novel (c.1695A>C) in 3’UTR region has been found in one individual. Other than these some already reported single nucleotide polymorphisms have also been identified in these 3 genes and some of them are more frequently found in CHD cases. In-silico analyses based on evolutionary, biochemical and structural aspect of the gene revealed the disease causing effect of these variants. Further biochemical analysis would reveal the regulatory circuits involving these cardiac transcription factors and their possible role in pathogenesis of the disease.

